# Mercury in human brain, blood, muscle and toenails in relation to exposure: an autopsy study

**DOI:** 10.1186/1476-069X-6-30

**Published:** 2007-10-11

**Authors:** Lars Björkman, Birgitte F Lundekvam, Torgils Lægreid, Bjørn I Bertelsen, Inge Morild, Peer Lilleng, Birger Lind, Brita Palm, Marie Vahter

**Affiliations:** 1 Dental Biomaterials Adverse Reaction Unit, Department of Health/UNIFOB, Årstadveien 17, NO-5009 Bergen, Norway; 2 Department of Oral Sciences, University of Bergen, Årstadveien 17, NO-5009 Bergen, Norway; 3 Department of Pathology, Haukeland University Hospital, NO-5021 Bergen, Norway; 4 Section for Pathology, The Gade Institute, University of Bergen, NO-5021 Bergen, Norway; 5 Institute of Environmental Medicine, Karolinska Institutet, PO Box 210, SE-171 77 Stockholm, Sweden

## Abstract

**Background:**

The main forms of mercury (Hg) exposure in the general population are methylmercury (MeHg) from seafood, inorganic mercury (I-Hg) from food, and mercury vapor (Hg^0^) from dental amalgam restorations. While the distribution of MeHg in the body is described by a one compartment model, the distribution of I-Hg after exposure to elemental mercury is more complex, and there is no biomarker for I-Hg in the brain. The aim of this study was to elucidate the relationships between on the one hand MeHg and I-Hg in human brain and other tissues, including blood, and on the other Hg exposure via dental amalgam in a fish-eating population. In addition, the use of blood and toenails as biological indicator media for inorganic and organic mercury (MeHg) in the tissues was evaluated.

**Methods:**

Samples of blood, brain (occipital lobe cortex), pituitary, thyroid, abdominal muscle and toenails were collected at autopsy of 30 deceased individuals, age from 47 to 91 years of age. Concentrations of total-Hg and I-Hg in blood and brain cortex were determined by cold vapor atomic fluorescence spectrometry and total-Hg in other tissues by sector field inductively coupled plasma-mass spectrometry (ICP-SFMS).

**Results:**

The median concentrations of MeHg (total-Hg minus I-Hg) and I-Hg in blood were 2.2 and 1.0 μg/L, and in occipital lobe cortex 4 and 5 μg/kg, respectively. There was a significant correlation between MeHg in blood and occipital cortex. Also, total-Hg in toenails correlated with MeHg in both blood and occipital lobe. I-Hg in both blood and occipital cortex, as well as total-Hg in pituitary and thyroid were strongly associated with the number of dental amalgam surfaces at the time of death.

**Conclusion:**

In a fish-eating population, intake of MeHg via the diet has a marked impact on the MeHg concentration in the brain, while exposure to dental amalgam restorations increases the I-Hg concentrations in the brain. Discrimination between mercury species is necessary to evaluate the impact on Hg in the brain of various sources of exposure, in particular, dental amalgam exposure.

## Background

Most people are to some degree exposed to mercury vapor via dental amalgam restorations, inorganic Hg^2+ ^via food, methylmercury (CH_3_Hg^+^) via fish and sea mammals and thimerosal in vaccines [1]. In addition, some people are exposed to mercury in occupational settings. Dental personnel constitute one of the largest groups with occupational exposure to elemental mercury. Exposure may also occur in the production of electrical devices (batteries, switches, and fluorescent light bulbs) and in the production of chlorine and sodium hydroxide using electrolysis [1]. From a toxicological point of view the MeHg exposure via seafood and mercury vapor is the most important.

For decades the potential health risks from mercury exposure from dental amalgam restorations have been reviewed and evaluated [2-6]. It is well known that amalgam restorations continuously release elemental mercury vapor [7,8], which is inhaled and absorbed by the body and distributed to tissues including the brain [9-11]. While it is clear that MeHg easily passes the blood brain barrier leading to higher levels of MeHg in brain than in blood, it is generally not believed that mercury in blood reflects the concentrations of inorganic mercury in the brain [12]. From a diagnostic point of view it is important to find out to what extent mercury levels in blood and other tissues may reflect concentrations of inorganic mercury in brain in a population with exposure to both methylmercury from fish and elemental mercury vapor from dental amalgam.

The aim of the present study was to explore the relationships between inorganic and organic mercury in brain (occipital lobe cortex) and in blood and other tissues in individuals without occupational exposure to inorganic mercury and to evaluate the use of blood and toenails as biological indicator media for inorganic and organic mercury (MeHg) in brain and other tissues.

## Methods

### Study population and sampling

In conjunction with routine autopsies at the Department of Pathology and the Department for Forensic medicine, the Gade Institute, Haukeland University Hospital, Bergen, Norway, samples from occipital lobe, thyroid gland, pituitary gland, abdominal muscle, toenails and blood from *vena femoralis *and the heart were collected from 30 deceased individuals. All autopsies were carried out within 3 days after death. Occupational exposure to mercury was used as an exclusion criterion. An equal distribution of men and women was aimed at.

Oral examination was carried out in every case. All dental restorations were recorded. Tooth surfaces filled with amalgam, composites or gold, and number of metalloceramic crowns, partial or complete fixed and removable dentures, and missing teeth were noted. Information on current and previous use of tobacco was obtained from the present hospital record as well as information on alcohol abuse, diseases and medication.

The project protocol was approved by the Regional Committee for Medical Research Ethics in Western Norway (#173.02), reviewed in relation to privacy and license requirements according to the Personal Data Registers Act by the Norwegian Social Science Data Services. According to Norwegian legislation the study was approved and registered as a temporary biobank by the Ministry of Health. Permission for sending human tissue samples for analyses out of Norway was given by the Directorate for Health and Social Affairs.

### Sampling procedure

Duplicate samples of blood from *vena femoralis *were collected after removal of the intestines by squeezing the blood out of the vene into a beaker. The samples were transferred into glass tubes with heparin as anticoagulant (BD Vacutainer^® ^Trace Element Tube, sodium heparin 119 IU, Becton Dickinson, UK), mixed well and transferred into polypropylene tubes (Sarstedt AG Co., Germany). Samples of blood from the heart were collected by aspiration into a syringe using a stainless steel cannula before the heart was opened. The samples were then treated as blood from *vena femoralis*. In some cases it was not possible to aspirate blood from the heart. Duplicate samples (about 3–4 g each) were collected from the occipital lobe by use of an acid washed scalpel (10 % HNO_3_) to minimize contamination and transferred into polypropylene tubes (Sarstedt AG Co., Germany). Samples from abdominal muscle (n = 30), pituitary (n = 30), thyroid (n = 28) and toenails (n = 29) were also collected. Generally, nails were clipped prior to the autopsy, and only small samples with a length of about 1 mm were available for collection. Clippings were collected from all toenails using a stainless steel scissors. Samples between 7.6 to 68 mg were used for analysis. All samples were frozen the same day and stored at -80°C. Before analysis, samples from occipital lobe were placed on a hydrocarbon wax film (Parafilm M^®^, SPI Supplies Division, Structure Probe Inc., West Chester, PA, USA) and the gray matter (cortex) was separated from the white matter of the samples using acid washed instruments. Only the gray matter was analyzed for mercury.

### Analyses of inorganic and total mercury by CVAFS

Samples of blood (from *vena femoralis *and the heart) and occipital lobe cortex were sent to Karolinska Institutet for speciation analyses of mercury. Briefly, the samples were solubilized in a mixture of L-cysteine (1 %), NaOH (45 %), and NaCl (1 %). Tissue samples were heated to complete the solubilization. Subsequently the solubilized samples were subjected to reduction by SnCl_2 _(for the determination of inorganic mercury) or a mixture of CdCl_2 _and SnCl_2 _(for the determination of total mercury). Mercury was determined by an automated multiple injection cold vapor atomic fluorescence spectrometric system (CVAFS; Merlin, PSA 10.023; P.S. Analytical Ltd., Orpington, Kent, UK) as previously described for blood [13]. The instrumentation is described in detail elsewhere [14]. Concentration of MeHg in tissue was estimated by subtracting the I-Hg concentration from the total-Hg concentration. Mercury content was related to the wet weight of the sample.

### Analyses of mercury by ICP-SFMS

Samples of blood from *vena femoralis *and of occipital lobe cortex, abdominal muscle, thyroid, pituitary gland, and toenails were analyzed for total mercury by double-focusing, sector field inductively coupled plasma-mass spectrometry (ICP-SFMS). The samples were subjected to microwave-assisted digestion with nitric acid as described previously for blood [15,16], and other soft tissues [17] and nails [18,19] at a commercial laboratory (ALS Analytica AB, Luleå, Sweden) [20]. The toenails were not cleansed before analysis.

### Analytical quality control

Reference materials LUTS-1 (non defatted lobster hepatopancreas; National Research Council of Canada/Institute for National Measurement Standards), Seronorm 404107X and Seronorm 404108 (whole blood; SERO A/S, Asker, Norway) were analyzed together with the samples as described for the analyses of inorganic and total mercury. For the analytical quality control of the analyses by ICP-SFMS the reference materials LUTS-1, SRM 8414 (bovine muscle powder; NIST), SRM 1577a (bovine liver; NIST) were used. Duplicate samples of blood from *vena femoralis *were analyzed for total mercury both at Karolinska Institutet using CVAFS and at Analytica AB using ICP-SFMS giving an opportunity to compare the methods. Although it could be expected that the mercury concentrations in the duplicate samples from occipital lobe cortex were not identical, duplicate samples were analyzed for total mercury by both methods (CVAFS and ICP-SFMS). MeHg in the reference material LUTS-1 was in addition determined at Analytica AB after digestion in methanolic KOH, extraction and ethylation of MeHg followed by determination by coupled gas chromatography-inductively coupled plasma mass spectrometry (GC-ICPMS) [21].

### Statistical methods

Associations between concentrations of mercury in blood and tissues were evaluated statistically by correlational analyses. Multiple linear regression was used to evaluate the importance of background variables (age, gender) and exposure indicators (number of amalgam surfaces, MeHg in blood) on concentration of mercury concentration in tissue. A stepwise backward method was used, entering all independent variables (age, gender, number of amalgam surfaces, MeHg in blood) at the first step. In the following step(s) the variables were removed, one at the time, if the assigned p-value was found to be 0.10 or higher. SPSS 13.0 (SPSS Inc., Chicago, IL, USA.) was used for the calculations.

## Results

Tissue samples from 13 women and 17 men (overall mean age 68 years, range 47 to 91 years, standard deviation 11.8 years) were collected (Table 1). Scrutiny of the present hospital records did not in any case reveal current or previous occupational exposure to mercury. There were two cases with documented alcohol abuse and 10 had received blood transfusions during the last months. The individuals had on average 13.2 dental amalgam surfaces (range 0 to 50 surfaces).

**Table 1:** Demographic data on the individuals included in the study.

Case #	Gender	Age (years)	Smoking status ^a^	Alcohol abuse ^b^	Amalgam surfaces	Transfusion ^c^
1	Male	79	CS	Y	0	Y
2	Female	78	N/A	N	6	N
3	Male	65	CS	N	4	Y
4	Male	73	NS	N	0	Y
5	Male	71	CS	N	5	Y
6	Male	48	N/A	N	43	N
7	Male	67	FS	N	1	N
8	Male	63	FS	N	43	N
9	Male	61	N/A	N	13	N
10	Female	78	CS	N	11	N
11	Male	71	CS	Y	0	Y
12	Male	90	NS	N	0	N
13	Female	70	FS	N	0	N
14	Male	78	N/A	N	0	N
15	Male	71	CS	N	2	Y
16	Female	63	CS	N	24	Y
17	Female	49	NS	N	42	N
18	Male	49	CS	N	29	Y
19	Male	71	FS	N	11	Y
20	Male	65	N/A	N	12	Y
21	Male	79	CS	N	0	N
22	Male	47	CS	N	0	N
23	Female	62	FS	N	5	N
24	Female	54	N/A	N	50	N
25	Female	78	FS	N	0	N
26	Female	64	N/A	N	24	N
27	Female	56	NS	N	24	N
28 ^d^	Female	91	NS	N	13	N
29	Female	83	N/A	N	0	N
30	Female	68	N/A	N	34	N

a) CS: Current smoker; FS: Former smoker; NS: Non smoker; N/A: Data not available

b) As noted in the hospital record (Yes/No)

c) Blood transfusion the last three months as noted in the hospital record (Yes/No)

d) Previous occupational exposure to mercury (retired dental nurse)

The median concentrations of MeHg and I-Hg in blood were 2.2 and 1.0 μg/L, respectively (Table 2). At an average, the MeHg/total-Hg ratio in blood was 0.62 (SD 0.22, median 0.67) with a range from 0.18 to 0.95. Mercury in tissue samples was related to the wet weight of the sample. In occipital lobe cortex the median concentrations of MeHg and I-Hg were 4 and 5 μg/kg wet weight, respectively. In one of the samples from occipital cortex the concentration of I-Hg (164 μg/kg) was 9 times higher than the concentration of the second highest case and fulfilled the criteria of an "extreme outlier" from a statistical point of view [22] (more than the value of the 75th percentile plus three times the inter quartile range). In order to get additional information on this case (woman, age 91), historical hospital records were examined and it was found that she had been employed as a dental assistant earlier in life. Therefore, the associations between number of amalgam surfaces and concentration of total-Hg, MeHg and I-Hg in blood and tissues were analyzed both with and without this case (number 28, Table 1). Historical hospital records were not checked for the other cases.

**Table 2:** Results from determinations of methylmercury, inorganic mercury and total mercury in tissues.

Tissue	Analyte ^a^	Median	Mean ^b^	SD	Min	Max	10-percentile	90-percentile
Blood (v. femoralis) (μg/L)	T-Hg	3.3	5	5.3	0.9	27.4	1.4	12.5
	I-Hg	1.0	2.3	4.2	0.1	22.4	0.2	5.2
	MeHg	2.2	2.7	2.3	0.3	10.9	0.9	6.2
Occipital cortex (μg/kg)	T-Hg	12	18	32	1	181	5	28
	I-Hg	5	12	29	2	164	3	18
	MeHg	4	6	6	<D.L.^c^	28	1	16
Abdominal muscle (μg/kg)	T-Hg	3	5	6	0.3	29	0.9	12
Thyroid gland (n = 28) (μg/kg)	T-Hg	19	55	186	2.7	1000	5.1	47
Pituitary gland (μg/kg)	T-Hg	43	200	540	4.9	2900	12	460
Toenails (n = 29) (μg/kg)	T-Hg	236	280	214	64	856	67	624

a) T-Hg: Total mercury concentration; I-Hg: Inorganic mercury concentration; MeHg: Methylmercury concentration

b) Samples with concentrations below detection limit were given a value of half the detection limit of the actual analytical run

c) Detection Limit

Abdominal muscle contained generally less than 30 μg/kg mercury (median 3 μg/kg). Both thyroid and pituitary contained considerably higher mercury concentrations; the median values were 19 μg/kg and 43 μg/kg, respectively, and there was a considerable variation ranging to 1,000 and 2,900 μg/kg, respectively (Table 2). The highest median total-Hg concentration was found in toenails (median 236 μg/kg), although the variation was small; the highest value being just 3.6 times higher than the median. Contrary to blood from *vena femoralis*, samples from heart blood were highly inhomogeneous due to extensive coagulation and we did not consider the concentrations obtained in those samples as reliable. Thus, no data are presented for heart blood.

**Figure 1:**
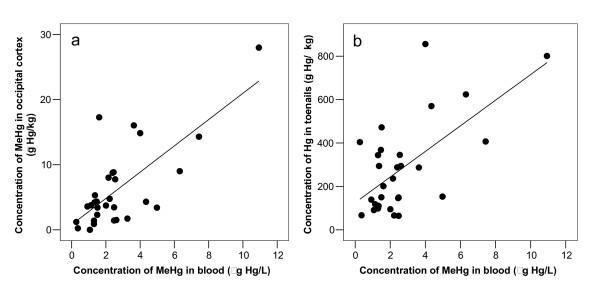
**Methylmercury in blood related to concentration of methylmercury in brain and total-Hg in toenails**. Concentration of methylmercury in blood (C_MeHg-Blood; μg Hg/L_) related to concentration of (a) methylmercury in occipital lobe cortex (C_MeHg-Brain; μg Hg/kg_) and (b) concentration of total-Hg in toenails (C_Hg-Toenail; μg Hg/kg_). The equations for the regression lines were (a) C_MeHg-Brain _= 2.0 × C_MeHg-blood _+ 0.8, (correlation coefficient 0.725, n = 30, p < 0.001) and (b) C_Hg-toenail _= 59 × C_MeHg-Blood _+ 125, (correlation coefficient 0.634, n = 29, p < 0.001).

**Figure 2:**
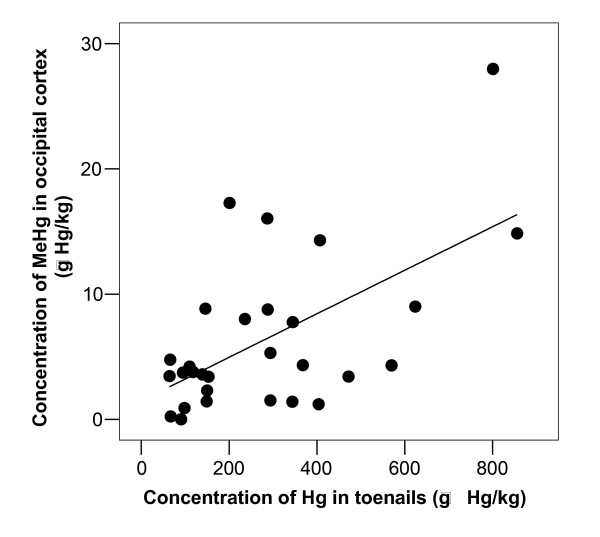
**Concentration of methylmercury in brain related to concentration of total-Hg in toenails**. Concentration of methylmercury in occipital lobe cortex (C_MeHg-Brain; μg Hg/kg_) related to concentration of total-Hg in toenails (C_Hg-Toenail; μg Hg/kg_). The equation for the regression line was C_MeHg-Brain _= 0.017 × C_Hg-toenail _+ 1.5, and the correlation coefficient was 0.586 (n = 29, p = 0.001).

Correlational analyses (Table 3) showed a significant association between MeHg in blood and brain cortex (Figure 1). Further, significant correlations were found between total-Hg in toenails and MeHg in blood (correlation coefficient 0.634, n = 29, p < 0.001; Figure 1) and occipital lobe cortex (correlation coefficient 0.586, n = 29, p = 0.001; Figure 2).

**Table 3:** Correlation coefficients between concentrations of mercury in tissues, age and number of amalgam surfaces. ^a^

		I-Hg in blood	MeHg in blood	I-Hg in occipital cortex	MeHg in occipital cortex	Hg in occipital cortex	Hg in pituitary	Hg in thyroid	Hg in toenail	Hg in abdominal muscle	Age	Filled surfaces – amalgam
I-Hg in blood	Correlation		0.251	-0.026	0.052	0.006	-0.062	-0.039	0.065	-0.045	**-0.402**	**0.432**
	Sig. (2-tailed)		0.181	0.891	0.784	0.974	0.744	0.845	0.738	0.815	0.028	0.017
	N		30	30	30	30	30	28	29	30	30	30
MeHg in blood	Correlation	0.246		-0.071	**0.725**	0.232	-0.107	-0.090	**0.634**	0.230	0.055	-0.126
	Sig. (2-tailed)	0.198		0.710	<0.001	0.218	0.572	0.648	<0.001	0.221	0.774	0.507
	N	29		30	30	30	30	28	29	30	30	30
I-Hg in occipital cortex	Correlation	0.253	0.134		0.339	**0.927**	**0.967**	**0.993**	-0.086	-0.155	0.334	0.081
	Sig. (2-tailed)	0.186	0.490		0.067	<0.001	<0.001	<0.001	0.656	0.414	0.071	0.669
	N	29	29		30	30	30	28	29	30	30	30
MeHg in occipital cortex	Correlation	0.079	**0.804**	0.081		**0.617**	0.287	0.336	**0.586**	0.258	0.154	-0.136
	Sig. (2-tailed)	0.686	<0.001	0.677		<0.001	0.124	0.081	0.001	0.169	0.416	0.472
	N	29	29	29		30	30	28	29	30	30	30
Hg in	Correlation	0.147	**0.713**	**0.604**	**0.768**		**0.904**	**0.910**	0.154	-0.048	0.295	0.080
occipital cortex	Sig. (2-tailed)	0.447	<0.001	0.001	<0.001		<0.001	<0.001	0.425	0.800	0.113	0.676
	N	29	29	29	29		30	28	29	30	30	30
Hg in pituitary	Correlation	-0.002	-0.062	**0.652**	-0.086	**0.390**		**0.953**	-0.100	-0.149	0.261	0.173
	Sig. (2-tailed)	0.993	0.748	<0.001	0.659	0.036		<0.001	0.607	0.432	0.163	0.360
	N	29	29	29	29	29		28	29	30	30	30
Hg in thyroid	Correlation	0.352	0.142	**0.602**	0.105	**0.396**	**0.402**		-0.087	-0.125	0.351	0.031
	Sig. (2-tailed)	0.072	0.479	0.001	0.603	0.041	0.038		0.667	0.525	0.067	0.875
	N	27	27	27	27	27	27		27	28	28	28
Hg in toenail	Correlation	0.060	**0.631**	-0.093	**0.649**	**0.525**	-0.095	-0.091		0.139	0.158	-0.328
	Sig. (2-tailed)	0.760	<0.001	0.638	<0.001	0.004	0.629	0.660		0.472	0.414	0.083
	N	28	28	28	28	28	28	26		29	29	29
Hg in abdominal muscle	Correlation	-0.055	0.220	-0.079	0.328	0.186	-0.038	0.335	0.130		-0.003	-0.081
	Sig. (2-tailed)	0.778	0.251	0.683	0.083	0.334	0.847	0.088	0.510		0.988	0.672
	N	29	29	29	29	29	29	27	28		30	30
Age	Correlation	**-0.408**	0.096	-0.208	0.037	-0.084	-0.287	-0.215	0.200	0.055		**-0.610**
	Sig. (2-tailed)	0.028	0.621	0.278	0.849	0.664	0.131	0.282	0.308	0.778		<0.001
	N	29	29	29	29	29	29	27	28	29		30
Filled surfaces – amalgam	Correlation	**0.433**	-0.127	**0.550**	-0.144	0.185	**0.541**	**0.530**	-0.328	-0.082	**-0.655**	
	Sig. (2-tailed)	0.019	0.513	0.002	0.457	0.337	0.002	0.004	0.088	0.673	<0.001	
	N	29	29	29	29	29	29	27	28	29	29	

^a ^The upper part of the table (above the diagonal) shows the correlations using all cases (n = 30) and the lower part (below the diagonal) shows the correlations after exclusion of the case with previous occupational exposure to mercury (dental nurse, see text). P-values and number of cases included in the analysis are given. Significant correlations (p < 0.05) in bold.

**Figure 3:**
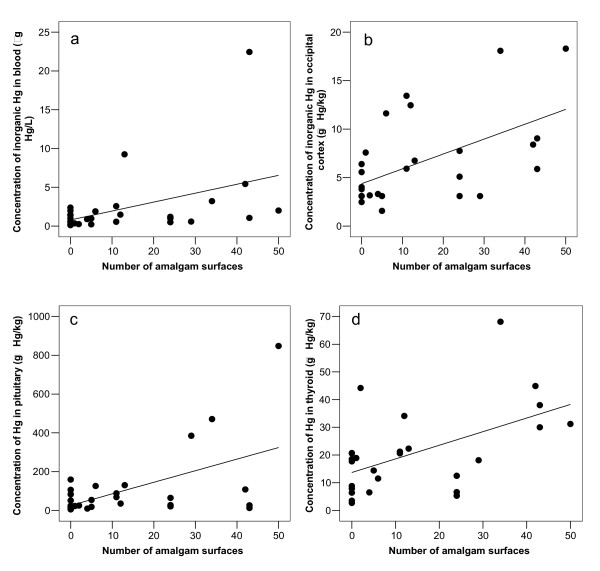
**Amalgam surfaces related to I-Hg in blood and brain, and total-Hg in pituitary and thyroid**. Number of amalgam surfaces related to concentration of (a) inorganic Hg in blood (C_I-Hg-Blood; μg Hg/L_), (b) inorganic Hg in occipital cortex (C_I-Hg-Brain; μg Hg/kg_), (c) total-Hg in pituitary (C_Pituitary; μg Hg/kg_) and (d) total-Hg in thyroid (C_Thyroid; μg Hg/kg_). The equations for the regression lines were (a) C_I-Hg-Blood _= 0.12 × [number of amalgam surfaces] + 0.79, (correlation coefficient 0.433, n = 29, p = 0.019), (b) C_I-Hg-Brain _= 0.15 × [number of amalgam surfaces] + 4.4, (correlation coefficient 0.550, n = 29, p = 0.002), (c) C_Pituitary _= 6.0 × [number of amalgam surfaces] + 25.5, (correlation coefficient 0.541, n = 29, p = 0.002), and (d) C_Thyroid _= 0.49 × [number of amalgam surfaces] + 13.7, (correlation coefficient 0.530, n = 27, p = 0.004).

There was a significant correlation between I-Hg in blood and the number of surfaces filled with dental amalgam at time of death (p = 0.019, Figure 3). Inclusion of the data from case 28 (the retired dental nurse) did not change this finding (Table 3). Both I-Hg in blood and number of surfaces filled with dental amalgam decreased significantly with age (Table 3). An increase in age by 10 years (from 50 to 60 years of age) was associated with a decrease in number of surfaces filled with dental amalgam, from 28.0 to 19.8 at the age of 60.

**Figure 4:**
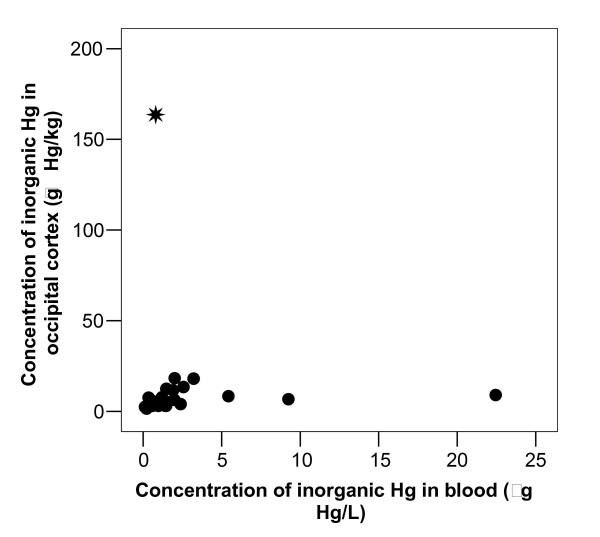
**I-Hg in brain related to I-Hg blood**. Concentration of inorganic Hg in occipital cortex related to concentration of inorganic Hg blood. The correlation coefficient was not significant (r = -0.026, p = 0.891, n = 30). When the dental nurse (case 28, denoted with  in the Figure) was excluded, the correlation coefficient was 0.253 (p = 0.186, n = 29).

**Figure 5:**
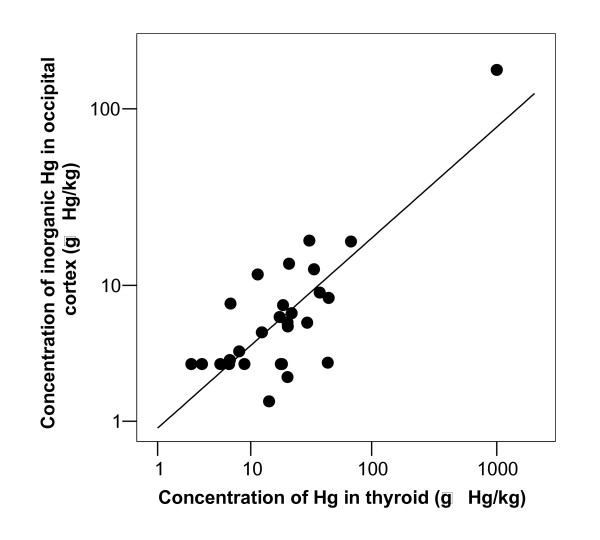
**I-Hg in brain related to concentration of total-Hg in thyroid**. Concentration of inorganic Hg in occipital cortex related to concentration of total-Hg in thyroid gland. The correlation coefficient of the log_10 _transformed values was 0.769 (n = 28, p < 0.001).

**Figure 6:**
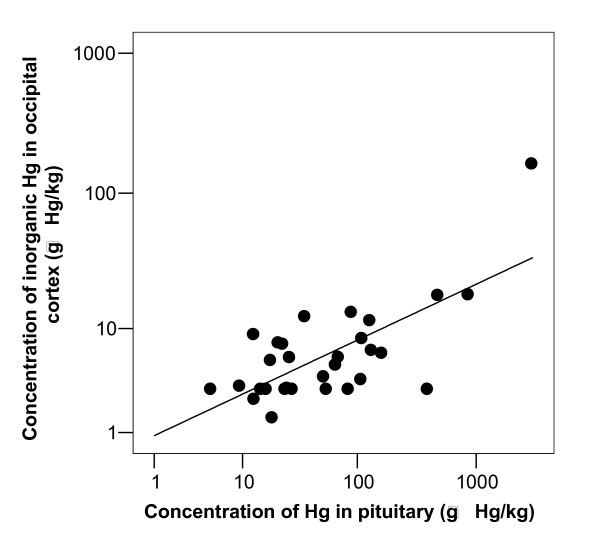
**I-Hg in brain related to total-Hg in pituitary**. Concentration of inorganic Hg in occipital cortex related to concentration of total-Hg in pituitary. The correlation coefficient of the log_10 _transformed values was 0.693 (n = 30, p < 0.001).

After exclusion of the retired dental nurse (case 28) the correlation coefficients between number of amalgam surfaces at time of death and concentration of I-Hg in occipital cortex, pituitary and thyroid were highly significant (Figure 3). The correlation coefficients were 0.55 (p = 0.002, n = 29), 0.54 (p = 0.002, n = 29) and 0.53 (p = 0.004, n = 27), respectively (Table 3). There was no significant association between I-Hg in blood and occipital lobe cortex (r = -0.026, p = 0.891, n = 30; Table 3 and Figure 4). I-Hg in occipital lobe cortex was significantly correlated with total mercury in thyroid and pituitary (Table 3, Figure 5 and 6).

Using the number of teeth filled with dental amalgam as a substitute for number of tooth surfaces filled with dental amalgam as estimate of amalgam exposure, similar results with only minor variations of the correlation coefficients were found. Nor was there any major change in the correlation coefficients when a more detailed measure of amalgam exposure ("amalgam points") [23] was used instead of number of surfaces filled with dental amalgam. The correlation coefficient between I-Hg in occipital lobe cortex and number of teeth filled with dental amalgam was 0.577 (p = 0.001, n = 29) and the correlation coefficient between I-Hg in occipital lobe cortex and "amalgam points" was 0.552 (p = 0.002, n = 29).

Analysis by linear multiple regression (stepwise, backward) with I-Hg in brain cortex as dependent variable and number of surfaces filled with amalgam, age, gender, and MeHg in blood as independent variables (excluding case 28 with previous occupational exposure to mercury) showed a significant effect from exposure to amalgam restorations (p = 0.002, Table 4). The regression coefficient of 0.153 (95 % confidence interval 0.061 to 0.245) indicated an average increase of about 1.5 μg I-Hg/kg brain cortex for each 10 amalgam filled surfaces. The other independent variables (age, gender, and MeHg in blood) were not significant in the model (Table 4).

**Table 4:** Results from linear multiple regression analyses. ^a^

Dependent variable	Independent variable(s) in the final model	Unstandardized Coefficients		Standardized Coefficients	t	Sig.	95 % Confidence Interval for B	
					
		B	Std. Error	Beta			Lower Bound	Upper Bound
a) I-Hg in brain cortex	Constant	4.366	0.925		4.720	<0.001	2.468	6.264
	Filled surfaces – amalgam	0.153	0.045	0.550	3.423	0.002	0.061	0.245
b) Total-Hg in pituitary	Constant	25.540	36.893		0.692	0.495	-50.158	101.238
	Filled surfaces – amalgam	5.972	1.788	0.541	3.340	0.002	2.304	9.640
c) Total-Hg in thyroid	Constant	13.682	3.359		4.073	<0.001	6.763	20.601
	Filled surfaces – amalgam	0.491	0.157	0.530	3.126	0.004	0.168	0.815
d) Total-Hg in toenails	Constant	126.115	50.564		2.494	.019	22.180	230.051
	MeHg in blood (μg/L)	58.888	14.190	0.631	4.150	<0.001	29.720	88.056

^a ^Final results from linear multiple regression analyses using number of surfaces filled with amalgam, age, gender, and MeHg in blood as independent variables (all variables entered at the first step, and removed in the following step(s) if the p-value of the variable was ≥0.10) and (a) I-Hg in brain cortex (μg I-Hg/kg) as dependent variable, (b) Total-Hg in pituitary (μg Hg/kg) as dependent variable, (c) Total-Hg in thyroid (μg Hg/kg) as dependent variable and (d) Total-Hg in toenails (μg Hg/kg) as dependent variable. The case with previous occupational exposure to mercury (case 28) was excluded from the regression analyses (see text). N = 29 in model (a) and (b), n = 27 in model (c) and n = 28 in model (d).

Similar regression models were used for analyses of total mercury in pituitary (n = 29), thyroid (n = 27) and toenail (n = 28). The results showed significant effects from number of surfaces filled with amalgam for the models for pituitary and thyroid. In the model for total-Hg in toenail, only MeHg in blood was significant (Table 4).

**Figure 7:**
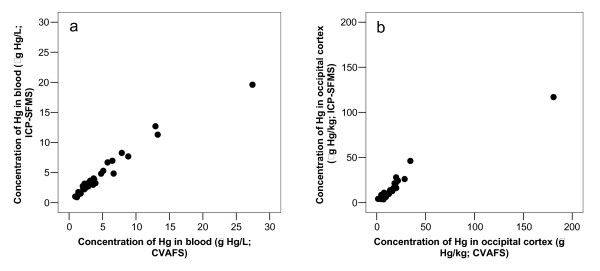
**Determinations of total-Hg by ICP-SFMS related to determinations of total-Hg by CVAFS**. Data from determinations of total-Hg by ICP-SFMS related to determinations of total-Hg by CVAFS in duplicate samples of blood (a) and occipital cortex (b). Correlation coefficients were 0.979 (n = 30) and 0.971 (n = 30), respectively.

Results from the analytical quality control are given in Table 5. Scatterplots of data from determinations of Hg by CVAFS and ICP-SFMS in duplicate samples of blood and occipital cortex are shown in Figure 7.

**Table 5:** Results from the analytical quality control

		Concentrations found				
			
Material	Analyte ^a^	Mean	SD	N	Method	Recommended (or certified) values
LUTS-1; ng/g	T-Hg	16.1	-	1	CVAFS	19.98 (0.20) ^b^
	I-Hg	14.1	-	1	CVAFS	-
	MeHg ^d^	2.0	-	1	CVAFS	2.50 (0.21) ^b^
LUTS-1; ng/g	MeHg ^d^	2.7	-	1	GC-ICPMS	2.50 (0.21) ^b^
Seronorm 404107X; μg/L	T-Hg	2.5	0.14	3	CVAFS	3 (2.2–3.3) ^c^
	I-Hg	0.5	0.03	3	CVAFS	-
	MeHg ^d^	2.0	0.13	3	CVAFS	-
Seronorm 404108; μg/L	T-Hg	8.5	0.74	4	CVAFS	8 (6.7–8.4) ^c^
	I-Hg	6.3	0.18	4	CVAFS	-
	MeHg ^d^	2.2	0.68	4	CVAFS	-
LUTS-1; ng/g	T-Hg	19.9	1.19	12	ICP-SFMS	19.98 (0.20) ^b^
SRM 8414; ng/g	T-Hg	5.5	0.63	8	ICP-SFMS	5 ± 3^e^
SRM 1577a; ng/g	T-Hg	3.6	0.51	8	ICP-SFMS	4 ± 2^e^

a) T-Hg: Total mercury concentration; I-Hg: Inorganic mercury concentration; MeHg: Methylmercury concentration

b) Mean and one standard deviation, n = 4 for Hg and n = 5 for MeHg (Revised value by February 14, 2005; written personal communication, Dr. Ralph Sturgeon, National Research Council of Canada/Institute for National Measurement Standards). Values from the certificate, revised in August 1995, were 16.7 for T-Hg and 9.4 for MeHg with 95 percent confidence limits of 2.2 and 0.6, respectively.

c) Recommended value (range)

d) as Hg

e) Estimated uncertainties

## Discussion

This study provides support to the importance of dental amalgam as a source of I-Hg in both blood and brain. In addition, total-Hg concentration in pituitary and thyroid was strongly associated with the number of dental amalgam surfaces. However, there is no evidence from epidemiological studies that exposure to dental amalgam restorations is associated with disease, impaired neuropsychological functions or prevalence of general health complaints [24-29]. Nevertheless, an association between amalgam exposure and subclinical neurological effects have been reported [30], which may be consistent with an association between cumulative amalgam exposure and some neurological diagnoses as reported by Bates et al. [28].

Although the number of amalgam surfaces at the time of death do not reflect lifetime cumulative exposure to Hg^0 ^or dental amalgam restorations, there was a significant correlation between amalgam surfaces at the time of death and concentration of I-Hg in brain at the time of death. This suggests that the biological half-time in the brain of the main fraction of Hg stored in brain after low level Hg^0 ^exposure from dental amalgam restorations and other low level environmental sources is relatively short and that other environmental sources have limited importance at a group level.

As expected, we found a positive association between MeHg in blood and brain cortex. Interestingly, we also found a significant correlation between total mercury in toenail on the one hand and MeHg in blood and brain cortex on the other. The toenail clippings were collected from all toes, reflecting an integrated measure of incorporated mercury between 100 and 140 days before death, based on a mean toenail growth rate of 0.07 mm/day [31]. Thus, toenail clippings of about 1 mm would more closely reflect blood levels at a time window of about two weeks; 3 to 5 months prior to the clipping, and the correlation should be interpreted with this in mind. Toenail mercury has, like hair mercury, been used in several studies as indicator of exposure and in order to get a better understanding of the relations between MeHg exposure and in MeHg in blood and toenail the lag time should be considered. There was no significant association between total mercury in toenail and I-Hg, which fits with the assumption that mercury in toenails, like that in hair [14], is likely to be almost exclusively MeHg. Thus, total mercury in toenails may be an indicator of MeHg stored in the body, and may be a useful complement to mercury in hair for MeHg exposure assessment [32]. The ratio of total Hg in nail to MeHg in blood was about 100, which is less than one third of the hair to blood ratio [14].

A small fraction of MeHg in the brain demethylates to inorganic mercury, which is retained for very long times, especially in pituitary and thalamus [33], probably mainly due to association with selenium to highly insoluble mercury selenide [34]. Consequently, a fraction of the inorganic mercury in brain could be related to past exposure to MeHg. Possibly, the absence of an increase in I-Hg in occipital cortex by age was due to the relatively low exposure to methylmercury in this population [35,36]. Because of the relatively low MeHg exposure the MeHg level in blood was low, and consequently the MeHg/total-Hg ratio in blood was low compared with populations with high exposure to MeHg and low exposure to I-Hg [37]. However, there was a wide variation in the individual MeHg/total-Hg ratio in blood indicating that the group was heterogeneous with regard to MeHg exposure, fish intake and exposure to I-Hg.

It is well known that drugs may be redistributed within hours after death, which may result in erroneous *post mortem *values in blood [38]. In the present study, the median concentration of total-Hg in blood was similar to levels found in Norwegian populations [39,40]. Thus, the use of blood from *vena femoralis *collected *post mortem *is likely a useful medium for monitoring recent exposure to mercury. The samples of blood from the heart were not homogenous and could not be used to evaluate possible post mortal redistributions of mercury.

Despite the fact that the concentrations of I-Hg in both blood and brain cortex were significantly correlated with the number of dental surfaces with amalgam fillings, there was no significant correlation between I-Hg in blood and brain cortex (Table 3 and Figure 4). This may be explained by the differences in toxicokinetics between elemental mercury (Hg^0^) and oxidized mercury. Although elemental mercury is rapidly oxidized by catalase in the blood to mercuric mercury (Hg^2+^), a substantial fraction of the inhaled elemental mercury will pass the blood brain barrier before being oxidized. Probably, most of the mercury vapor is then oxidized to mercuric mercury (Hg^2+^) in the brain. Mercuric mercury does not pass the blood brain barrier and, consequently, it is not distributed as such into the brain. Amalgam fillings not only release elemental mercury vapor, but they also corrode and release corrosion products [41]. These corrosion products contain mercuric mercury and other metals (*e.g*. silver), which are swallowed and partly absorbed in the GI tract into blood [42]. Individual variation in exposure to corrosion products released from amalgam fillings may contribute to the lack of correlation between inorganic Hg in blood and inorganic Hg in brain in addition to the differences in toxicokinetics of I-Hg in blood and brain.

One individual (case 6) had a relatively high I-Hg in blood (22.4 μg/L), constituting about 82 % of the total blood Hg. This individual had a high number of dental amalgam restorations (43 amalgam surfaces), but there were no obvious signs of bruxism or intense chewing which could explain the high level of I-Hg in blood [43]. In conflict with the high level of I-Hg in blood, the concentration of I-Hg in brain of this case (9 μg/kg) was in the same range as for the rest of the group without previous occupational exposure to mercury. An explanation could be that the corrosion rate of the amalgam restorations was elevated and thus, the gastrointestinal exposure to amalgam corrosion products was higher than normal, although the exposure to mercury vapor derived from amalgam restorations was in the normal range. This was the only case from the Department for Forensic medicine, and another possibility is that movement of the body after death may have influenced *post mortem *redistribution of mercury [38].

There was a significant correlation between I-Hg in brain cortex and the number of amalgam surfaces, but only 30 % of the variance was explained by the regression model (Figure 3; r = 0.55, r^2 ^= 0.30). This may in part be due to variations between individuals regarding release rate of mercury vapor from dental amalgam, and the toxicokinetic variations between individuals with regard to elemental mercury vapor [8,10,44,45]. Further, additional variation could be due to demethylated Hg derived from MeHg in the brain [33]. A possible effect from variations in selenium status on I-Hg in brain needs further analyses.

We found one case with considerably higher brain inorganic mercury concentration than the others. Although, occupational exposure to mercury was an exclusion criterium, when the study was designed, it was first when we carefully examined the historical hospital records that we found out that this case was a retired dental nurse. She was the oldest individual in the study group and had probably been working as a dental assistant at times when the mercury exposure in dental clinics in Norway was considerably higher than today [46]. As the exposure to mercury in dental personnel in Norway was relatively high in the 1960ies, with reported median concentration of mercury in urine among dental nurses of 32 μg/L urine (160 nmol/L) with a range from 1.5 μg/L to 620 μg/L (7.5 to 3100 nmol/L) [47], exclusion of this case was justified.

It is well known that occupational exposure to high concentrations of mercury vapor can result in high levels of mercury in brain and other tissues several decades after the cessation of the exposure [48-50], indicating long half-times for inorganic mercury stored in the brain after exposure to high levels of mercury vapor. However, in conflict with data reported by Weiner and Nylander [51] the regression model analyzing the effects from amalgam surfaces, age, gender, and MeHg in blood on the concentrations of I-Hg in brain (excluding the dental nurse; Table 4) showed no significant influence by age, suggesting that the major fraction of mercury derived from amalgam restorations has a relatively short half-time in brain. However, the statistical power was too low to exclude a minor effect from age. The observations of high levels of I-Hg in brain several years after the cessation of occupational exposure to high levels of mercury vapor, but no significant effect from age in the regression analysis (Table 4), suggests that the kinetics of I-Hg stored in brain after exposure to mercury vapor may be dose dependent with an induction of an alternative pathway for deposition of I-Hg in the brain. One hypothesis is that when the exposure to mercury vapor is low, Hg is bound to sites with low total capacity, but with higher affinity than to a proposed selenium metabolite forming highly insoluble HgSe with long biological half-time in the brain (see above) [52]. Alternatively, there is a mercury induced selenium dependent mechanism resulting in a formation of highly insoluble mercury selenide which could explain the observations.

The concentrations of total mercury in occipital cortex found in the remaining cases, without known occupational exposure to mercury, were similar to the concentrations of total mercury earlier reported for deceased individuals without occupational exposure to mercury [9], but lower than those reported by Saxe et al [53]. A significant correlation between mercury in brain and number of surfaces filled with amalgam is reported previously [9]. However, Saxe et al [53] found no significant correlation between exposure to dental amalgam and total mercury in brain, which may be due to the impact from MeHg in the diet, which not was considered.

It is previously reported that mercury is stored in the pituitary of dental personnel [49]. In the present study significant correlations between number of surfaces filled with amalgam and mercury concentrations in pituitary and thyroid. Probably, mercury is stored together with selenium in these tissues, resulting in very long biological half times [54]. High concentrations of I-Hg in pituitary were also reported after experimental exposure of monkeys to MeHg [33] and there was some evidence for a co-accumulation of I-Hg with selenium [55].

There were no significant correlations between mercury concentration in abdominal muscle and mercury concentration in any of the other tissues, and the hypothesis that muscle biopsy could be used to predict mercury concentrations in the brain [56] was not supported.

The quality control of the mercury analyses indicated acceptable results. The problems with the stability of MeHg in reference materials stored for long times [57] was illustrated with the results from the analyses of the reference material LUTS-1. Obviously, there was a break down of MeHg to I-Hg over time, resulting in a significant decrease of MeHg in the reference material without a decrease of total-Hg in the sample. Consequently, Hg and MeHg were removed from the certificate of LUTS-1 in the revision of January 2005 [58].

## Conclusion

In a fish-eating population, MeHg from the diet has a marked impact on total mercury concentration in the brain. Discrimination between mercury species is thus necessary to evaluate impact of dental amalgam exposure as well as fish consumption on Hg concentrations in the brain.

MeHg in blood is a useful marker for MeHg in brain. Total-Hg in toenails is a convenient and useful biomarker for MeHg in brain.

There were no useful biomarkers for I-Hg in the brain, but for individuals without occupational exposure to mercury, the number of tooth surfaces filled with amalgam was an indicator of concentration of I-Hg in brain at time of death, and a useful exposure indicator at a group level.

## Abbreviations

CH_3_Hg^+ ^Methylmercury

CVAFS Cold vapor atomic fluorescence spectrophotometry

GC-ICPMS Coupled gas chromatography-inductively coupled plasma mass spectrometry

Hg Mercury

Hg^0 ^Elemental mercury

Hg^2+ ^Mercuric mercury

ICP-SFMS Sector field inductively coupled plasma-mass spectrometry

I-Hg Inorganic mercury

IU International unit

MeHg Methylmercury

NIST National Institute of Standards and Technology

SD Standard deviation

SRM Standard Reference Material

## Competing interests

The author(s) declare that they have no competing interests.

## Authors' contributions

LB conceived of the study and participated in its design and coordination, performed the statistical analysis, and drafted the manuscript. BFL participated in the study design, performed the sample collection, participated in the dental examinations and sample preparation, and contributed to the preparation of the manuscript. TL participated in the study design, performed the sample collection and dental examinations, participated in the sample preparation, and contributed particularly to the Methods section of the manuscript. BIB assisted in the study design, provided expert advice on post mortal tissue sampling and contributed to the preparation of the manuscript. IM assisted in the study design, provided expert advice on post mortal tissue sampling and aided in the preparation of the manuscript. PL assisted in the study design, provided expert advice on post mortal tissue sampling and contributed to the preparation of the manuscript. BL assisted in the study design, provided expert advice on trace element analysis and analytical quality control and contributed to the preparation of the manuscript. BP performed the trace element speciation analysis and aided in the preparation of the manuscript. MV assisted in the study design, provided expert advice and contributed to the preparation of the manuscript.

All authors read and approved the final manuscript.
